# MJNMF-GAT: Multi-Task Joint Non-Negative Matrix Factorization Graph Attention Network for Understanding Adolescent Neurodevelopment

**DOI:** 10.1109/access.2025.3601649

**Published:** 2025-08-22

**Authors:** BINISH PATEL, TONY W. WILSON, JULIA M. STEPHEN, VINCE D. CALHOUN, YU-PING WANG

**Affiliations:** 1 Biomedical Engineering Department, Tulane University, New Orleans, LA 70118, USA; 2 Institute for Human Neuroscience, Boys Town National Research Hospital, Boys Town, NE 68010, USA; 3 Mind Research Network, Albuquerque, NM 87106, USA; 4 Tri-Institutional Center for Translational Research in Neuroimaging and Data Science (TReNDS), Georgia State University, Georgia Institute of Technology, Emory University, Atlanta, GA 30030, USA

**Keywords:** Age prediction, deep learning, fMRI, graph attention network, multi-task, neurodevelopment

## Abstract

Integration of multi-task brain imaging data is crucial for furthering our understanding of neurodevelopment. By utilizing functional magnetic resonance imaging (fMRI) data and machine learning methods such as graph neural networks to measure brain activity, one can efficiently capture complex brain network interactions. In this work, we introduce the Multi-Task Joint Non-Negative Matrix Factorization Graph Attention Network (MJNMF-GAT) to investigate fMRI data of multiple tasks with the goal of determining age-related differences in adolescence. Our framework integrates joint non-negative matrix factorization for extracting shared latent features across tasks with graph attention networks to model brain connectivity patterns. The model is capable of integrating data from different fMRI tasks to enhance both predictive performance and network interpretation. We applied GNNExplainer for model interpretability and identified key subnetworks that contribute to prediction tasks. The proposed MJNMF-GAT model demonstrates superior results over existing methods, achieving a root mean square error (RMSE) of 1.9212 ± 0.1742, mean absolute error (MAE) of 1.5368 ± 0.1469, and correlation of 0.8032 ± 0.0469 in an age prediction task on the Philadelphia Neurodevelomental Cohort (PNC). The model also identifies the critical functional connections that change with the different stages of adolescent neurodevelopment. Our approach improves the predictive accuracy and, more importantly, interprets informative brain connectivity patterns, especially for detecting those important sub-networks responsible in age prediction. The MJNMF-GAT framework opens a new avenue for analyzing multi-task neuroimaging studies, which helps identify significant brain networks and develop a deeper understanding of functional connectivity changes in adolescence.

## I. INTRODUCTION

The period of adolescence is a critical time for neurodevelopmental changes in the brain. It is a period characterized by significant changes in structure and functionality, which are necessary in the development and maturation of cognitive, emotional, and social capabilities. These neurodevelopmental changes and patterns have lasting effects through-out adulthood, which can influence behavior and mental health [1], [2], [4], [4]. From the lens of neurodiversity, adolescence is also when individual variations in brain development become more evident, illustrating the extensive range of neurological diversity within the population. Neurodivergence, which includes conditions such as autism, ADHD, and dyslexia, often becomes more pronounced during this period due to the brain’s rapid adaptation to genetic and environmental influences [5]. Thus, the study of brain anatomy and function during adolescence is essential for neuroscientists to deepen their understanding of various factors, including structural neurodevelopment, brain maturation, cognitive advancements, and the specific brain regions involved in these processes. This insight is valuable for identifying typical developmental trajectories and identifying neurodivergent individuals, who may need support for tailored approaches in education and healthcare [6].

Machine learning techniques, especially graph neural networks (GNNs), provide a useful tool in the analysis of fMRI data in order to find new biomarkers associated with developmental differences associated with age, gender, and cognition. Employing GNNs allows researchers to reveal hidden patterns and relationships in fMRI data that were previously unknown, and thus offer new insights into the complex interaction between age and brain activity [7]. These methods are also instrumental in exploring the neurobiological foundations of neurodivergence, helping to elucidate how different brain connectivity patterns correspond to various cognitive and behavioral profiles [8]. The present study is particularly important for enhancing our knowledge regarding how different neurodivergent brains work.

Blood oxygen level-dependent functional magnetic resonance imaging (BOLD fMRI) is a non-invasive imaging method that allows a far greater understanding of neurodevelopmental changes taking place in the human brain while attaining transition into adulthood [9]. Functional MRI data can provide information during task performance and rest. Data from the Philadelphia Neurodevelopmental Cohort (PNC) [10] provides two different types of fMRI datasets, one collected during a working memory paradigm known as nback fMRI and another during an emotion recognition paradigm, which is referred to as emoid fMRI. These fMRI tasks provide the opportunity to compute functional connectivity measures that quantify the relationships between different brain regions. A common approach to obtaining these measures is through Pearson correlation coefficients derived from the time series data of various brain areas. Both the raw fMRI data and the computed functional connectivity data are high dimensional [11]. The number of variables is usually very large compared to the number of subjects. Moreover, the variables are often highly inter-correlated, which makes analysis and interpretation challenging [12].

These challenges become quite relevant if one deals with a broad spectrum of brain functioning in diverse populations. For instance, individuals with autism may display unique connectivity patterns that differ from those of neurotypical individuals, especially in brain regions linked to social cognition and sensory processing [13]. Consequently, applying advanced machine learning techniques like GNNs to such complex data not only enhances our understanding of typical neurodevelopment but also helps in identifying the distinct neural characteristics associated with neurodivergence. This can lead to more customized and effective interventions [14]. The use of multimodal data to predict brain age has shown to be more effective than approaches that focus on a single modality [15], [16]. An example of this is Cole et al. [15], where the authors propose a novel method that employs multimodal multiple regression analysis using various MRI modalities from a group of healthy subjects. They argue that integrating multiple predictors allows for a more comprehensive understanding of age-related changes in brain tissue. Additionally, their findings support the prediction of age in different brain regions [16]. By considering the interaction between multiple data sources, a better understanding of the complexity of the brain can be grasped, particularly in the neurodivergent individuals. This may allow for an elucidation of how specific brain regions or networks are uniquely contributing to experiences of neurodivergent individuals [17].

The rest of the manuscript is organized as follows. Section II provides an overview of related work to our proposed method. Section III discusses a review of the joint non-negative matrix factorization method, graph attention network, and introduces the proposed MJNMF-GAT framework. In Section IV, the experimental results are presented with ablation studies and a comparison of our method with alternative frameworks. Section V includes a detailed discussion of the analysis of the PNC data to shed light on the different stages of adolescence. Lastly, Section VI concludes the manuscript with a discussion regarding its limitations.

## II. RELATED WORK

Non-Negative Matrix Factorization (NMF) has been widely utilized in fMRI analysis to uncover latent structures within multimodal datasets. Anderson et al. [18] employed NMF to differentiate ADHD from healthy controls by identifying changes in default mode subnetworks. However, the study’s reliance on a single modality limits its ability to capture complex interactions across multiple data types, potentially overlooking critical aspects of brain function. Similarly, Sotiras et al. [19] demonstrated that NMF can generate sparse, parts-based representations aligned with the brain’s functional organization. While effective, this approach primarily focuses on structural covariance and may not fully account for functional connectivity or dynamic brain processes.

Advanced NMF methods have sought to address these limitations by handling more complex, multimodal data. Peng et al. [20] introduced a Group Sparse Joint Non-Negative Matrix Factorization (JNMF) framework, integrating multiple modalities such as fMRI to identify risk genes and abnormal brain regions associated with schizophrenia. While this method enhances interpretability, it may still struggle with the high dimensionality and heterogeneity of multimodal data, potentially leading to less robust findings when applied to diverse or noisy datasets.

Graph-based methods integrated with NMF have also been proposed to model complex relationships in neuroimaging data. Zhu et al. [21] presented a Graph Regularized NMF (GNMF) method for cancer gene clustering, which improves robustness by capturing the geometric structure of the data space. However, the GNMF’s application to neuroimaging data may be limited by its sensitivity to the choice of graph construction parameters, which can significantly impact the quality of the clustering results. Wang and Ma [22] further combined NMF with network embedding for graph co-clustering, enabling simultaneous clustering of nodes and edges in a graph. This approach, while innovative, may face challenges in scalability and computational efficiency, particularly when applied to large neuroimaging datasets with complex graph structures.

Deep learning approaches combined with NMF offer additional potential but come with their own set of limitations. Moon and Lee [23] introduced the Joint Deep Semi-Non-Negative Matrix Factorization (JDSNMF) model, which captures shared latent features in multi-omics data through hierarchical, non-linear feature extraction. Although this method can uncover complex patterns in brain connectivity, it may be prone to overfitting, particularly in cases where the data is sparse or lacks sufficient training examples, which can limit its generalizability to new datasets.

The integration of NMF with graph attention networks (GATs) is an emerging area of research that shows promise for fMRI analysis. Sjolund and Bankestad [24] discussed a graph-based neural acceleration method for NMF that leverages the relationship between matrices and bipartite graphs, making it suitable for large-scale neuroimaging data. However, the challenge lies in balancing computational efficiency with the preservation of important graph structures, as aggressive acceleration techniques might inadvertently discard valuable information.

Li et al. addressed some of these challenges by introducing a spatially constrained NMF method for fMRI data analysis, which enhances the identification of relevant brain network components by incorporating spatial prior information into the NMF objective function [25]. Despite its advancements, this method is limited by its reliance on accurate spatial priors, which may not always be available or reliable, potentially leading to biased results.

These studies collectively highlight both the potential and the limitations of current methods, paving the way for the development of a Multi-Task Joint Non-Negative Matrix Factorization Graph Attention Network (MJNMF-GAT) for fMRI studies. The proposed MJNMF-GAT framework aims to address these limitations by integrating NMF with graph attention mechanisms, enhancing the extraction of meaningful brain connectivity patterns from multi-task fMRI data. This integration holds promise for more precise and interpretable neuroimaging analyses. The model is robust to the challenges of high-dimensional as well as noisy data, and it can scale effectively to large and complex datasets.

## III. METHODOLOGY

### A. OVERVIEW OF PIPELINE

**FIGURE 1.**
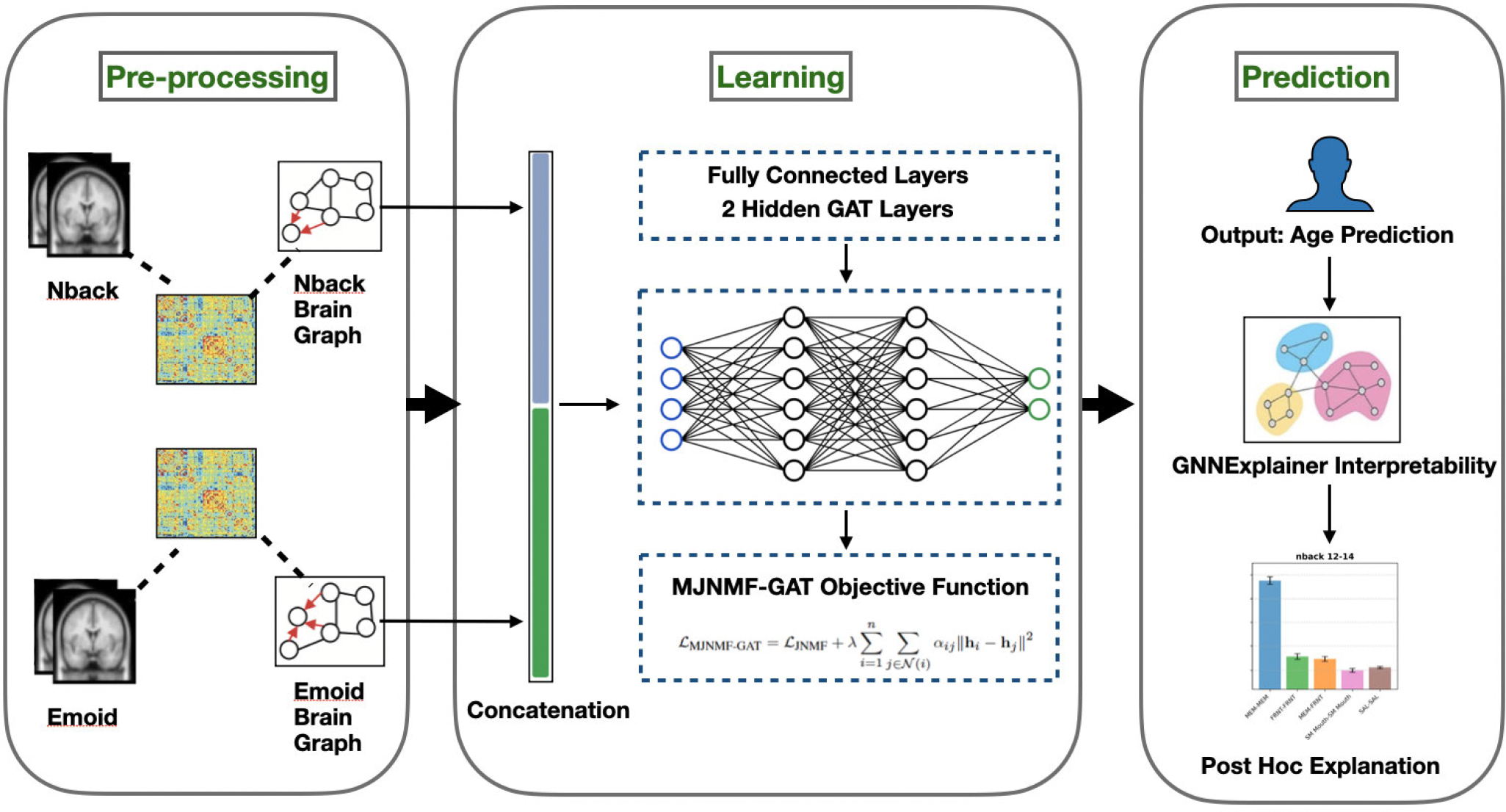
Overview of the pipeline.

Fig. 1 provides an overview of the analytical approach employed in this study. For each participant, the fMRI time-series data underwent pre-processing, after which the entire brain was segmented into 264 regions of interest (ROIs) using the Power coordinates template [26]. The mean BOLD time series was extracted from each brain region, and the Pearson correlation coefficient between the ROIs was computed, resulting in a functional connectivity matrix. The brain graph for both emoid and nback data was concatenated. Then the data was placed into our proposed Multi-Task Joint Non-negative Matrix Factorization Graph Attention Network model to predict age based on the different stages of adolescence. Finally, the GNNExplainer method was utilized to pinpoint critical subnetwork structures and identify significant connections within the functional networks [27].

### B. DATA COLLECTION AND PRE-PROCESSING

The dataset utilized in this study was sourced from the Philadelphia Neurodevelopmental Cohort (PNC), a collaborative effort between the Children’s Hospital of Philadelphia and the University of Pennsylvania, funded by the National Institute of Mental Health (NIMH). The PNC aims to characterize interactions between brain function and behavior, involving over 800 healthy participants aged 8 to 22 years. The study employed fMRI data focused on emoid and nback tasks. All MRI scans were conducted on a single 3T Siemens TIM Trio whole-body scanner. During the tasks, participants were instructed to categorize emotions depicted in facial expressions, including happiness, anger, sadness, fear, and neutrality. The scanning session lasted for a total of 10.5 minutes, with the BOLD signal captured using a whole-brain, single-shot, multislice, gradient-echo echoplanar sequence consisting of 124 volumes [28].

Standard pre-processing procedures were carried out using SPM12, which included motion correction, spatial normalization to MNI standard space, and spatial smoothing using an 8 mm FWHM Gaussian kernel. Motion correction was applied to reduce the impact of head movement on functional connectivity estimates. While we recognize the importance of mitigating physiological noise sources such as respiratory and cardiac artifacts, we did not explicitly perform physiological noise correction, as physiological recordings were not available for all participants. Additionally, global signal regression (GSR) was not applied in this study, as GSR can potentially introduce spurious anti-correlations and alter the network topology. Our approach is consistent with previous studies that omit GSR to preserve intrinsic correlation structure. This pre-processing pipeline aligns with widely used protocols for functional connectivity analysis in developmental neuroimaging [28], [29].

**FIGURE 2.**
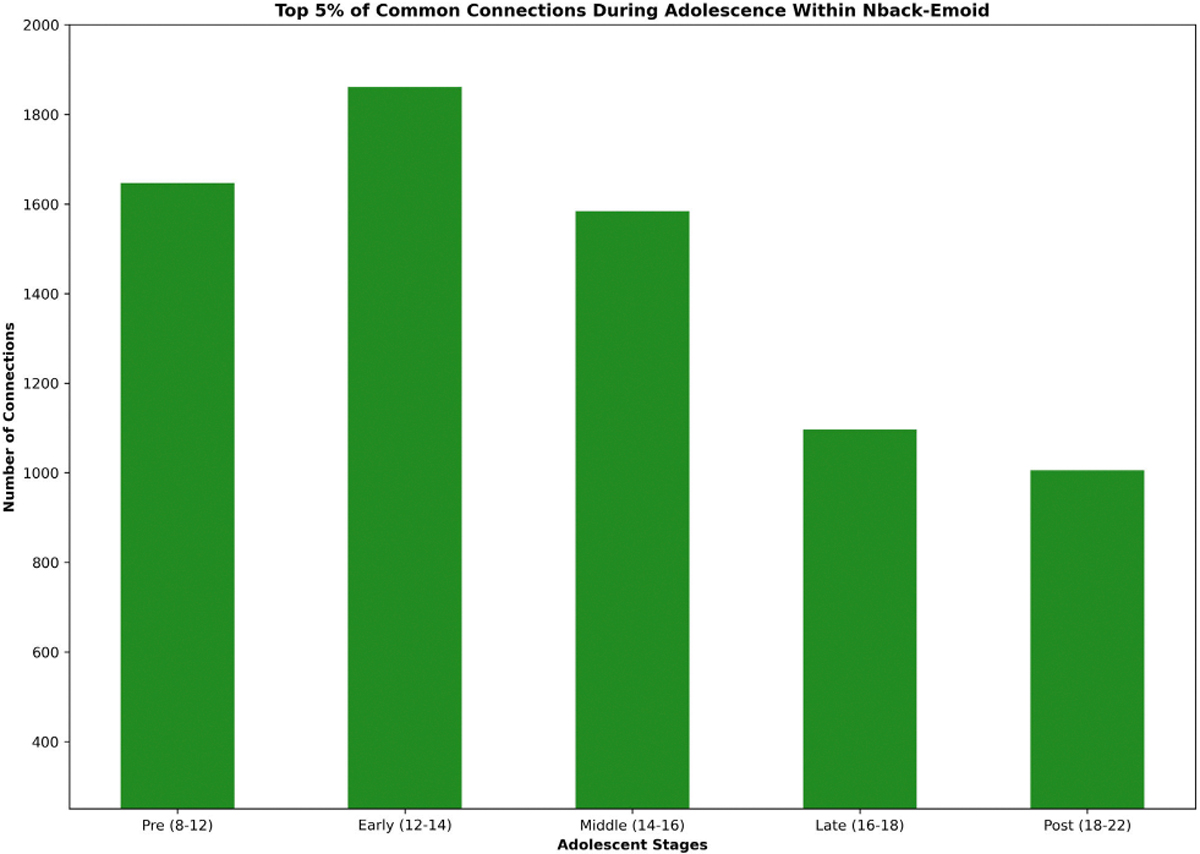
The distribution of subjects in the various stages of adolescence.

The Power template [26] was employed to divide the brain into 264 ROIs for the parcellation of the BOLD signal and the construction of the connectivity matrix using Pearson correlation. For this study, a subset of 622 participants was selected from the total pool of 800, chosen based on the availability of both the emoid and nback fMRI paradigms. The subjects were further divided into five groups, each representing a distinct stage of adolescent development, as depicted in Fig. 2 [29].

### C. JOINT NON-NEGATIVE MATRIX FACTORIZATION

Joint Non-Negative Matrix Factorization (JNMF) is a powerful technique used for analyzing and extracting meaningful patterns from fMRI data. JNMF is particularly effective in the context of fMRI because it allows for the simultaneous factorization of multiple related data matrices while enforcing non-negativity constraints. This technique is beneficial for identifying latent factors or brain networks that are consistent across different types of fMRI data or experimental conditions.

Consider K different non-negative matrices $X^{1},X^{2},\ldots ,X^{K}\in R^{N\times M}$, where N represents the number of brain regions (nodes) and M represents the number of observations or time points. The goal of JNMF is to decompose these matrices into a common set of non-negative factors that capture the underlying structure of the data. The joint factorization can be formulated as:

(1) $$X^{k}\approx W^{k}H^{k},\;\text{for}\;k=1,\;2,\ldots ,K$$

where $W^{k}\in R^{N\times R}$ is the matrix of non-negative basis vectors for the k-th dataset, $H^{k}\in R^{R\times M}$ is the matrix of non-negative coefficients, and R is the number of latent factors (or components). To enforce consistency across datasets, JNMF introduces a shared basis matrix $W\in R^{N\times R}$, and decomposes each matrix $X^{k}$ as:

(2) $$X^{k}\approx WH^{k}$$

where $H^{k}$ is specific to each dataset, but W is common across all datasets. The objective function for JNMF is to minimize the reconstruction error while enforcing non-negativity constraints on W and $H^{k}$. The objective function can be expressed as:

(3) $$ℒ=\sum_{k=1}^{K}{\left\|X^{k}-WH^{k}\right\|}_{F}^{2}$$

subject to:

$$W\geq 0,\;H^{k}\geq 0$$

where $\|\cdot {\|}_{F}$ denotes the Frobenius norm, which measures the difference between the observed and reconstructed data matrices. In fMRI studies, JNMF can be applied to multiple fMRI datasets to extract common spatial patterns of brain activity that are consistent across different experimental conditions or subjects. For example, JNMF can be used to identify common brain networks activated during various cognitive tasks or to compare brain network connectivity patterns between healthy and clinical populations. By jointly analyzing multiple fMRI datasets, JNMF helps in understanding the shared underlying brain networks and how these networks are modulated by different tasks or conditions [22], [23]. This approach can improve the robustness of the findings and provide a more comprehensive view of the brain’s functional organization. JNMF provides a framework for jointly analyzing multiple fMRI datasets while enforcing non-negativity constraints, leading to the identification of consistent latent factors across different experimental conditions [30]. This technique enhances the ability to uncover meaningful patterns in brain activity and connectivity, ultimately contributing to a better understanding of cognitive and clinical phenomena.

### D. GRAPH ATTENTION NETWORK

GATs have emerged as a powerful tool for analyzing fMRI data, particularly in the context of understanding the intricate functional connectivity patterns within the brain. GATs extend the capabilities of traditional Graph Convolutional Networks (GCNs) by incorporating an attention mechanism that allows the model to weigh the importance of different neighboring nodes adaptively, thereby capturing more nuanced relationships within the graph structure.

In fMRI analysis, the brain can be represented as a graph where nodes correspond to brain regions, and edges represent functional connections between these regions, typically measured through correlation or coherence of the fMRI signals. The application of GATs in this context enables the model to learn from the data more effectively by focusing on the most relevant connections, which is particularly useful given the noise and variability inherent in fMRI data. Let 𝒢=(𝒱,ℰ) denote a graph where 𝒱 represents the set of nodes (brain regions) and ℰ represents the set of edges (functional connections). Each node $v_{i}\in 𝒱$ is associated with a feature vector $h_{i}\in R^{F}$, where F is the number of features (e.g., fMRI time series or other region-specific attributes). The key innovation of GATs lies in the attention mechanism, which assigns a weight to each edge based on the features of the connected nodes. For a node $v_{i}$, the attention coefficient for a neighboring node $v_{j}$ is computed as:

(4) $$e_{ij}=LeakyReLU\left(a^{⊤}\left[Wh_{i}\;\|\;Wh_{j}\right]\right)$$

where $W\in R^{F^{\prime }\times F}$ is a learnable weight matrix, $a\in R^{2F^{\prime }}$ is a learnable attention vector, ‖ denotes the concatenation operation, and LeakyReLU is a non-linear activation function. The attention coefficients are then normalized across all neighbors of node $v_{i}$ using the softmax function:

(5) $$\alpha_{ij}={softmax}_{j}\left(e_{ij}\right)=\frac{exp\left(e_{ij}\right)}{\sum_{k\in 𝒩(i)}exp\left(e_{ik}\right)}$$

where 𝒩(i) denotes the set of neighbors of node $v_{i}$. Finally, the output feature vector for node $v_{i}$ is computed as a weighted sum of the transformed features of its neighbors:

(6) $$h_{i}^{\prime }=\sigma \left(\sum_{j\in 𝒩(i)}\alpha_{ij}Wh_{j}\right)$$

where σ is an activation function, such as LeakyReLU. When applied to fMRI data, GATs can effectively capture the dynamic and context-dependent nature of brain connectivity by allowing the network to emphasize connections that are most relevant to the task or condition under study. This approach is particularly useful for tasks such as brain state classification, identifying biomarkers of neurological conditions, or understanding the neural basis of cognitive processes [31], [32]. The ability of GATs to adaptively focus on the most informative connections makes them well-suited for handling the high-dimensional and noisy nature of fMRI data, ultimately leading to more accurate and interpretable models of brain function.

### E. MULTI-TASK NON-NEGATIVE MATRIX FACTORIZATION GRAPH ATTENTION NETWORK

Multi-Task Joint Non-Negative Matrix Factorization Graph Attention Network (MJNMF-GAT) represents an advanced approach for analyzing fMRI data by integrating the strengths of Joint Non-Negative Matrix Factorization (JNMF) with GATs. This methodology is designed to handle the complex nature of fMRI data and enhance the interpretability of functional connectivity patterns through the incorporation of attention mechanisms and factorization techniques.

The MJNMF-GAT framework combines the capabilities of JNMF for decomposing multiple related data matrices with GATs to capture intricate dependencies within the brain’s functional connectivity graph. This approach allows for the simultaneous analysis of different modalities or conditions while leveraging attention mechanisms to focus on the most relevant connections between brain regions.

Consider K different non-negative matrices $X^{1},X^{2},\ldots ,X^{K}\in R^{N\times M}$, where N represents the number of brain regions and M represents the number of observations or time points. The goal of MJNMF-GAT is to decompose these matrices into shared basis components that capture global connectivity patterns and subject or modality specific coefficients that encode individual variations. This decomposition facilitates extraction of biologically meaningful features that serve as inputs to the GAT.

The joint factorization with shared basis matrix W can be expressed as:

(7) $$X^{k}\approx {WH}^{k},\;\text{for}\;k=1,\;2,\ldots ,K$$

where $W\in R^{N\times R}$ is the shared non-negative basis matrix, and $H^{k}\in R^{R\times M}$ is the coefficient matrix specific to the k-th dataset. Each input matrix $X^{k}$ is obtained by thresholding the subject’s functional connectivity matrix to retain only positive correlations, ensuring compatibility with the non-negativity constraint. The objective function to minimize is:

(8) $${ℒ}_{\mathrm{JNMF}}=\sum_{k=1}^{K}{\left\|X^{k}-{WH}^{k}\right\|}_{F}^{2}$$

subject to:

$$W\geq 0,\;H^{k}\geq 0$$

To incorporate GATs, we model the brain connectivity as a graph 𝒢=(𝒱,ℰ) with nodes 𝒱 representing brain regions and edges ℰ representing functional connections. The attention mechanism allows the network to adaptively weigh the importance of edges between nodes. The attention coefficient $e_{ij}$ for an edge between nodes $v_{i}$ and $v_{j}$ is computed as:

(9) $$e_{ij}=LeakyReLU\left(a^{⊤}\left[Wh_{i}\;\|\;Wh_{j}\right]\right)$$

where $W\in R^{F^{\prime }\times F}$ is a learnable weight matrix, $a\in R^{2F^{\prime }}$ is a learnable attention vector, and LeakyReLU is an activation function. The attention coefficients are normalized using the softmax function. Importantly, while W is shared across subjects and modalities to represent common connectivity patterns, the subject-specific coefficient matrices $H^{k}$ provide individualized node features. These features, combined via W, serve as personalized inputs to the GAT, enabling subject-specific prediction despite the shared basis.

The MJNMF-GAT model integrates JNMF and GAT to simultaneously factorize multi-task fMRI data and refine brain connectivity representations. The shared basis matrix W obtained from JNMF is used as input to the GAT, which then applies attention mechanisms to emphasize the most critical connections between brain regions. The combined objective function for MJNMF-GAT is:

(10) $${ℒ}_{\text{MJNMF-GAT}}={ℒ}_{JNMF}+\lambda \sum_{i=1}^{n}\sum_{j\in 𝒩(i)}\alpha_{ij}{\left\|h_{i}-h_{j}\right\|}^{2}+\mu {ℒ}_{\text{pred}}$$

The total objective function for MJNMF-GAT combines three components: a reconstruction loss from JNMF, a smoothness regularization term, and a prediction loss that guides age regression. Specifically, the final loss function is given in Equation 10, where λ and μ are regularization coefficients that balance the contribution of the smoothness and prediction terms, respectively. The coefficient λ controls the strength of the local smoothness constraint by penalizing the dissimilarity between latent representations of neighboring nodes. A higher value of λ enforces stronger smoothness constraints, which can improve generalization and reduce overfitting by promoting more coherent node embeddings across the graph. Conversely, a lower λ allows the model to focus more on task-specific (i.e., age prediction) objectives. The term ${\left\|h_{i}-h_{j}\right\|}^{2}$ in the loss function denotes the squared Euclidean distance between the latent node embeddings $h_{i}$ and $h_{j}$, encouraging local smoothness by penalizing differences between connected nodes. Meanwhile, the prediction loss is captured by ${ℒ}_{\text{pred}}$ and defined as the mean squared error (MSE) between the predicted and actual ages. The coefficient μ modulates the influence of this term, ensuring that the learned representations are optimized not only for structural coherence but also for accurate age prediction.

MJNMF-GAT is particularly well-suited for analyzing fMRI data across different conditions or tasks, such as task-based fMRI. By jointly modeling these datasets, the method can reveal consistent and distinctive connectivity patterns associated with different cognitive states or behaviors. The attention mechanism further enhances interpretability by highlighting the most relevant brain connections, which can serve as potential biomarkers for various neurological conditions.

### F. GNNEXPLAINER FOR INTERPRETABILITY

Additionally, we utilized GNNExplainer to interpret our model and identify key functional connectivity subnetworks that were crucial in age prediction. GNNExplainer is a model-agnostic method designed to provide explanations for the classification outcomes of GNN-based models [27]. This interpretability approach offers consistent insights into the significant node features that contribute critically to the prediction. To determine the most important node features, GNNExplainer treats $X_{FS}$ as a subset of features and learns a feature selector F, which serves as a feature mask. This mask is applied to the nodes in the explanation of $G_{s}$ defined by the feature selector.

The explanation ($G_{s},X_{s}$) is simultaneously optimized to maximize the mutual information objective of ($G_{s},F$), which reflects the modified objective function that combines structural and node feature data to clarify the prediction $\overset{ˆ}{y}$ at node v [30]. The hyperparameters used in this method include prediction loss, feature size loss, feature element loss, population size loss, population element loss, weight decay, training epochs, and learning rate, with respective values of 1, 200, 20, 0, 1000, 0, 150, and 5 × 10^−1^ as detailed in Table 2. Upon applying GNNExplainer and analyzing the significant subnetworks, we further identified common connections associated with age prediction within each subnetwork. The GNNExplainer method uses five loss functions: prediction loss, feature size loss, feature element loss, population size loss, and population element loss. Prediction loss, typically binary cross-entropy, measures the error between predicted and true labels. Feature size and element losses penalize changes to node features, while population size and element losses penalize changes to graph size and edge weights, respectively. Together, these losses guide the algorithm to identify key features and edges influencing the prediction [27].

### G. EXPERIMENTAL DESIGN AND AGE PREDICTION

In this study, the dataset was randomly partitioned into training, validation, and test sets using an 80%/10%/10% split. To ensure balanced representation across age groups, we employed stratified sampling based on age bins during the splitting process. This approach mitigates the risk of age-related imbalances and ensures that each subset reflects the overall age distribution of the dataset, which is critical for robust and generalizable brain age prediction. The model was trained on the training set, while the hyperparameters were optimized using the validation set. Model performance was assessed by calculating root mean square error (RMSE), mean absolute error (MAE), and Pearson correlation coefficient, each reported with their corresponding standard deviation (std) across 10 repeated experiments. To aid interpretation, we contextualize MAE and RMSE relative to the dataset’s age range of 8–22 years. For example, a reported MAE of approximately 1.5 years constitutes less than 11% of the full age span, suggesting high predictive precision. Each experiment involved splitting the dataset, training the model, and performing testing. We compared the results of our proposed model with those of other approaches using the outcomes of these repeated experiments. Pairwise t-tests were performed, and p-values were reported to indicate any statistically significant improvements. To ensure statistical robustness and account for variability across runs while mitigating sampling bias, we employed bootstrapping over 10 repeated experiments. For each pairwise comparison, model performance metrics were resampled with replacement to generate empirical distributions of the test statistics, thereby reinforcing the stability of the significance estimates.

To ensure optimal performance and generalizability of the MJNMF-GAT model, we employed a systematic hyperparameter tuning procedure using a random search strategy. Specifically, key hyperparameters including the learning rate, weight decay, optimizer type, and number of training epochs were tuned with respect to model performance on the validation sets. This approach follows best practices outlined in prior studies, which demonstrate that random search can be more efficient and effective than grid search when exploring large hyperparameter spaces [33]. As detailed in Table 3, the hyperparameters for the experiments included the learning rate, optimizer, number of epochs, weight decay, fMRI paradigms, and the predictive task. The two-layer GATs described in Equation 1 were employed for the graph embedding process. The activation function for the two-layer MJNMF-GAT model was LeakyReLU, which was utilized to integrate the graph embeddings from the two modalities.

The learning rate was searched within the range of {1 × 10^−6^, 5 × 10^−6^, 1 × 10^−5^, 5 × 10^−5^, 1 × 10^−4^}, while weight decay values spanned {0.0, 0.1, 0.2, 0.3}. The number of epochs was varied between 1000 and 8000 to capture sufficient convergence without overfitting. The Adam optimizer was selected as the primary optimization algorithm, consistent with contemporary graph neural network training protocols. After conducting multiple random search iterations, the combination of a learning rate of 1 × 10^−5^, weight decay of 0.2, and 6000 epochs was found to consistently yield the best validation performance in terms of mean absolute error and correlation metrics.

We applied the MJNMF-GAT model to both working memory and emotion task fMRI data and compared its performance with other models. The results, including RMSE and MSE with corresponding p-values, are presented in Table 5. After evaluating our method’s performance, we conducted age prediction across the five stages of adolescence to determine whether our proposed framework could effectively utilize functional connectivity as brain fingerprints.

## IV. RESULTS

To enhance the performance of the MJNMF-GAT model, a systematic optimization of its hyperparameters was conducted using the random search method on dedicated validation sets [33]. The hyperparameters considered in the experiments encompassed several crucial aspects, namely the learning rate, optimizer, number of epochs, weight decay, fMRI paradigms, and predictive task. The specific values assigned to these hyperparameters were 1e-5 for the learning rate, Adam for the optimizer, 3000 for the number of epochs, 0.2 for the weight decay, and emoid/nback for the fMRI paradigms, while the predictive task focused on age prediction. To facilitate the process of graph embedding, a two-layer GAT architecture was employed. Following the graph attention layers, two fully connected (FC) layers were used to perform the final regression. These FC layers transform the learned node embeddings into a prediction output, allowing the model to map the graph-level representations to the target variable. The first FC layer applies a nonlinear transformation, while the second outputs the continuous prediction, completing the MJNMF-GAT prediction pipeline. The activation layers within the two-layer MJNMF-GAT model were set to ReLU, a commonly utilized activation function. This activation function was employed to merge the graph embeddings derived from the two modalities, ensuring their effective integration.

The assessment of age prediction was conducted using a rigorous 10-fold cross-validation approach, which was independently executed 10 times on the complete dataset. To determine the statistical significance of the classification outcomes achieved by our MJNMF-GAT model in comparison to other competing models, p-values were computed through a t-test on the results of repeated experiments. Our findings revealed that the integration of two fMRI paradigms, namely emoid and nback, resulted in superior performance when compared to the utilization of a single paradigm. This underscores the benefits of incorporating multi-paradigm fMRIs.

Utilizing GNNExplainer, we conducted an interpretive analysis of our model, enabling us to discern the critical subnetworks by identifying the top 5% of common functional connections. Both working memory and emotion tasks were employed for fMRI in this investigation. The functional networks were categorized using the following abbreviations: sensorimotor (SM Hand and SM Mouth), visual (VIS), default mode (DMN), fronto-parietal task control (FRNT), unknown (CB), and salience (SAL). The quantity of common functional connections varied across the five stages of adolescence. We found the specific ROIs of the most influential connections, comprising the top 5% for age prediction during adolescence. Displays the important intra and inter-network connections during the various stages of adolescence. Displays the count of top 5% common connections within and between MRI tasks at different developmental stages.

### A. ABLATION STUDIES

In our ablation studies, we evaluated the impact of the alpha parameter and NMF rank on the performance of the proposed MJNMF-GAT framework. Alpha is a regularization parameter used to control the complexity of the model and prevent overfitting. Specifically, it adds a regularization term to the objective function being minimized during factorization. Additionally, NMF rank refers to the number of latent features or components that the input matrix is factorized into. The NMF rank is critical because it determines the dimensionality of the reduced space and affects the interpretability and accuracy of the factorization.

As outlined in Table 4, we examined the model’s performance without JNMF and found it does not perform as well as when you include JNMF within the framework with a RMSE (2.1781 ± 0.2199), MAE (1.6688 ± 0.1792), and correlation (0.7392 ± 0.0592). When we investigated the effect of varying the alpha parameter, we found that the optimal configuration consists of alpha at 0.1, yielding a RMSE of 1.9914 ± 0.3261, MAE of 1.5640 ± 0.2693, correlation coefficient of 0.7825 ± 0.0509. Moreover, our experiments highlighted the influence of NMF rank on the MJNMF-GAT framework. The NMF rank of 5 was found to best yield the optimum performance with a RMSE of 1.9764 ± 0.0761, MAE of 1.5559 ± 0.2188, and correlation of 0.7862 ± 0.0509. Lastly, as presented in Table 4, we evaluated the performance of GAT across different modalities. The results indicated that the combined use of both emoid and nback fMRI modalities yielded the lowest metrics, with a RMSE of 2.1781 ± 0.2199, an MAE of 1.6688 ± 0.1792, and a correlation coefficient of 0.7392 ± 0.0592. A possible explanation for this phenomena is the increased dimensionality of data when combining both the emoid and nback paradigms.

### B. COMPARISON OF PERFORMANCE WITH ALTERNATIVE METHODS

The proposed MJNMF-GAT model demonstrated superior performance in age prediction when compared to several state-of-the-art algorithms, including LR, MLP, GCN [34], GAT [32], MPNN [35], ONMF [36], NMF-GCN, PCA-GCN [37], BrainNetCNN [38], BrainGNN [39] (refer to Table 5). MLP, a multi-layer perceptron, consists of multiple layers of neurons, each applying a non-linear activation function to the weighted sum of its inputs. GCN [34] is a deep learning architecture that applies convolutional operations to graph-structured data, aggregating information from neighboring nodes to capture local and global patterns, enabling tasks like node and graph classification, as well as link prediction. Moreover, GAT [32] is a deep learning framework that applies attention mechanisms to graph-structured data, allowing nodes to assign different importance weights to their neighbors during feature aggregation, enhancing the model’s ability to capture complex relationships and improve performance. NMF-GCN is a model that combines NMF with GCNs to extract meaningful latent features from non-negative data representations, facilitating the incorporation of these features into the GCN framework for improved performance. PCA-GCN [37] is a hybrid model that integrates Principal Component Analysis (PCA) with GCNs to reduce the dimensionality of input features before applying graph convolutions, thereby enhancing computational efficiency and preserving essential structural information. BrainNetCNN [38] is a deep learning architecture designed for brain connectivity matrices that leverages edge-to-node, node-to-graph, and edge-to-edge convolutions to model complex spatial patterns in brain networks for predictive tasks. BrainGNN [39] is a graph neural network framework tailored for fMRI analysis that integrates attention-based node pooling and interpretability modules to capture hierarchical and salient brain connectivity features for individual-level prediction. MJNMF-GAT, the proposed multi-task deep learning framework, was designed for neurodevelopmental studies to aid with dimensionality reduction and classification of heterogeneous data.

In terms of hyperparameter tuning, GAT model was validated using emoid and nback fMRI datasets with the same hyperparameter settings applied in the GAT component of MJNMF-GAT. For MLP, hyperparameters were optimized using the random search method. The MLP framework utilized the same network architecture as MJNMF-GAT, with specific hyperparameters including 4,000 training epochs, an L1 regularization parameter of 1e-2, an L2 regularization parameter of 1e-3, the Adam optimizer, weight decay of 0.2, and a learning rate of 2e-5. The NMF rank was 5 with an alpha parameter of 0.1. The prediction performance was evaluated using 10-fold cross-validation, repeated 10 times independently across the entire dataset. The p-values were calculated using a t-test to compare the classification results of MJNMF-GAT against other models.

To ensure reproducibility, functional connectivity matrices were z-scored and thresholded to retain the top 10% of connections. Dimensionality reduction used NMF with k=50 components (using W as node features) and PCA retaining the top 50 components. Graphs for NMF-GCN were constructed using Pearson correlations thresholded at 0.3, while PCA-GCN used a k=10 nearest neighbors graph in PCA space. Both models employed a two-layer GCN with 64 hidden units, LeakyReLU activation, and 0.5 dropout. Training used Adam optimizer with learning rate 1 × 10^−4^, weight decay 0.01, and up to 6000 epochs max.

**FIGURE 3.**
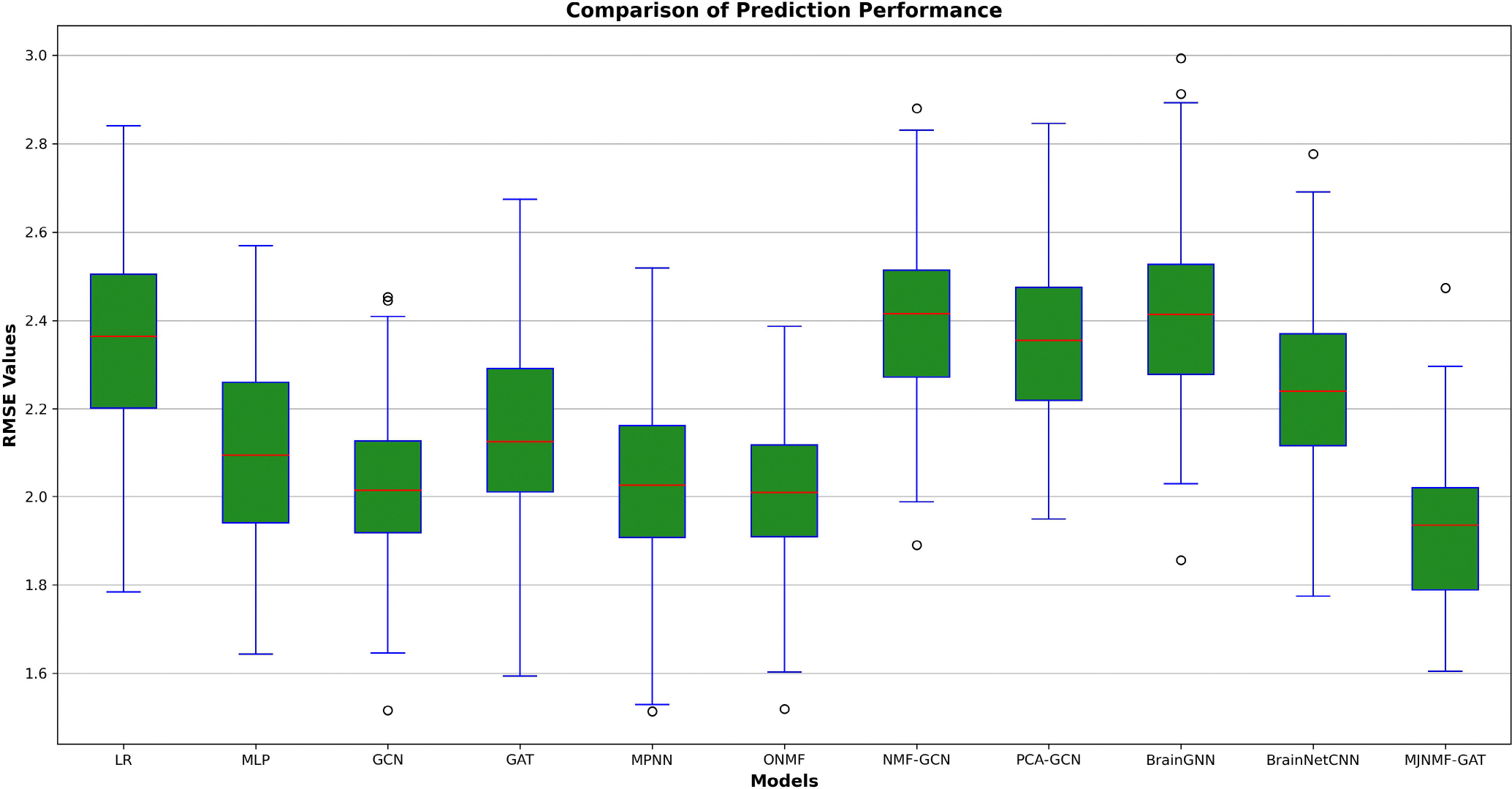
Prediction performance of different models.

Our findings indicated that integrating JNMF with the GAT framework resulted in higher performance compared to using simply GAT to predict age. Table 5 presents the performance of the nine algorithms tested on the PNC dataset. MJNMF-GAT performed best on the age prediction task with a RMSE at 1.9212 ± 0.1742, followed by MLP with 2.0901 ± 0.1845. As illustrated in Fig. 3, MJNMF-GAT outperformed the five other algorithms, and the results from the PNC dataset confirm that the integration of JNMF with GAT improves prediction performance.

### C. MODEL EXPLANATION AND BIOMARKER IDENTIFICATION

We analyzed and interpreted our model using GNNExplainer to identify the top 5% of significant functional connections, highlighting important subnetworks for both working memory and emotion task fMRI data. The functional networks investigated included sensorimotor (SM Hand and SM Mouth), visual (VIS), default mode (DMN), fronto-parietal task control (FRNT), cerebellum (CB), and salience (SAL). The number of common functional connections fluctuated across the five stages of adolescence. Table 6 presents the top 0.1% of key connections relevant to age prediction during adolescence, while Fig. 6 provides a visualization of these key regions of interest in axial, coronal, and sagittal views. Additionally, Fig. 7 displays the distribution of the top 5% common connections both within and across fMRI tasks at different stages of development. Meanwhile, Fig. 8 illustrates a heatmap visualization of the top 5% of common connections in each task fMRI.

## V. DISCUSSION

**FIGURE 5.**
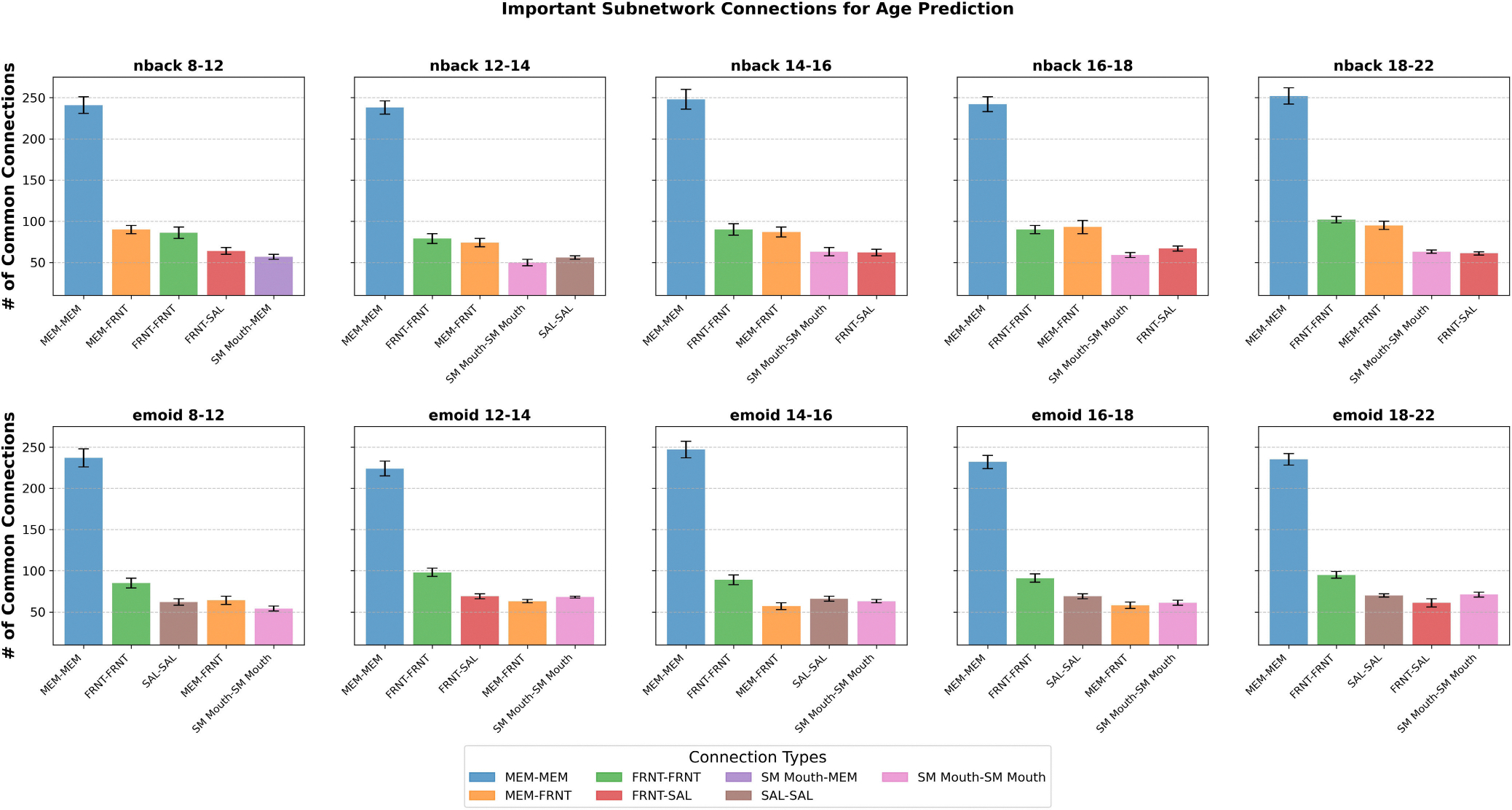
Important subnetwork connections during each stage of adolescence.

**FIGURE 6.**
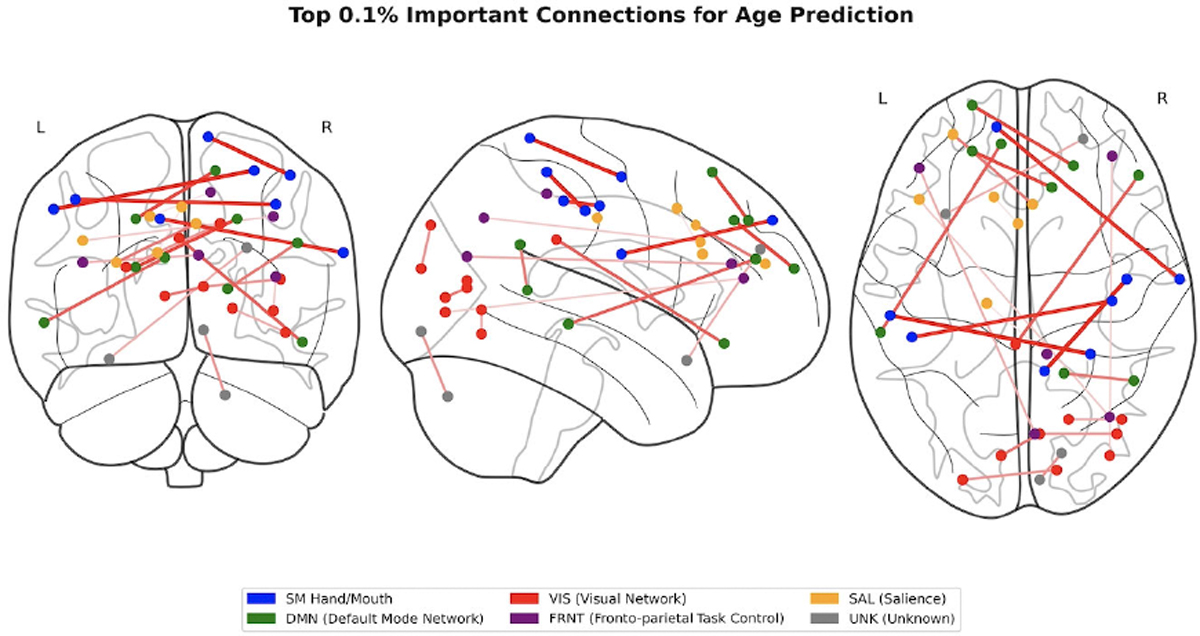
Top 0.1% of important connections in age prediction.

**FIGURE 7.**
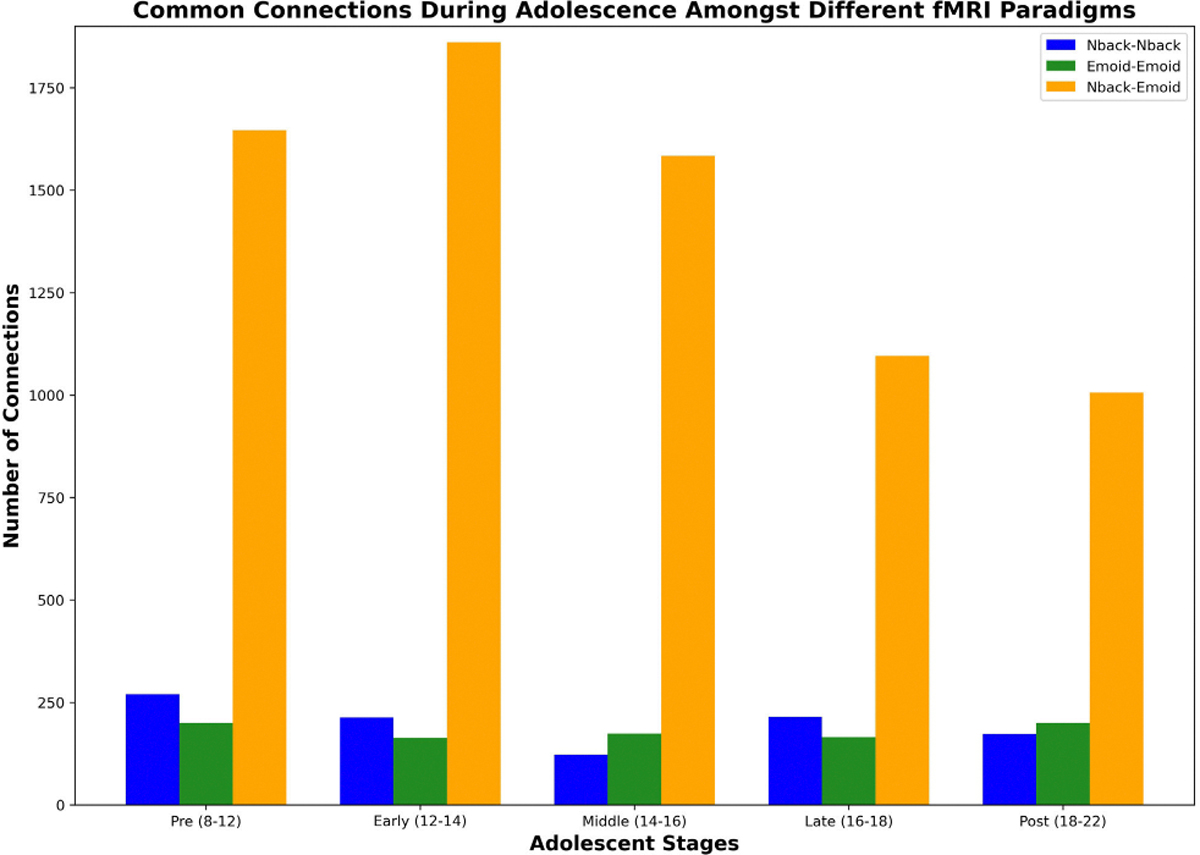
Top 5% number of common connections within and between MRI tasks.

**FIGURE 8.**
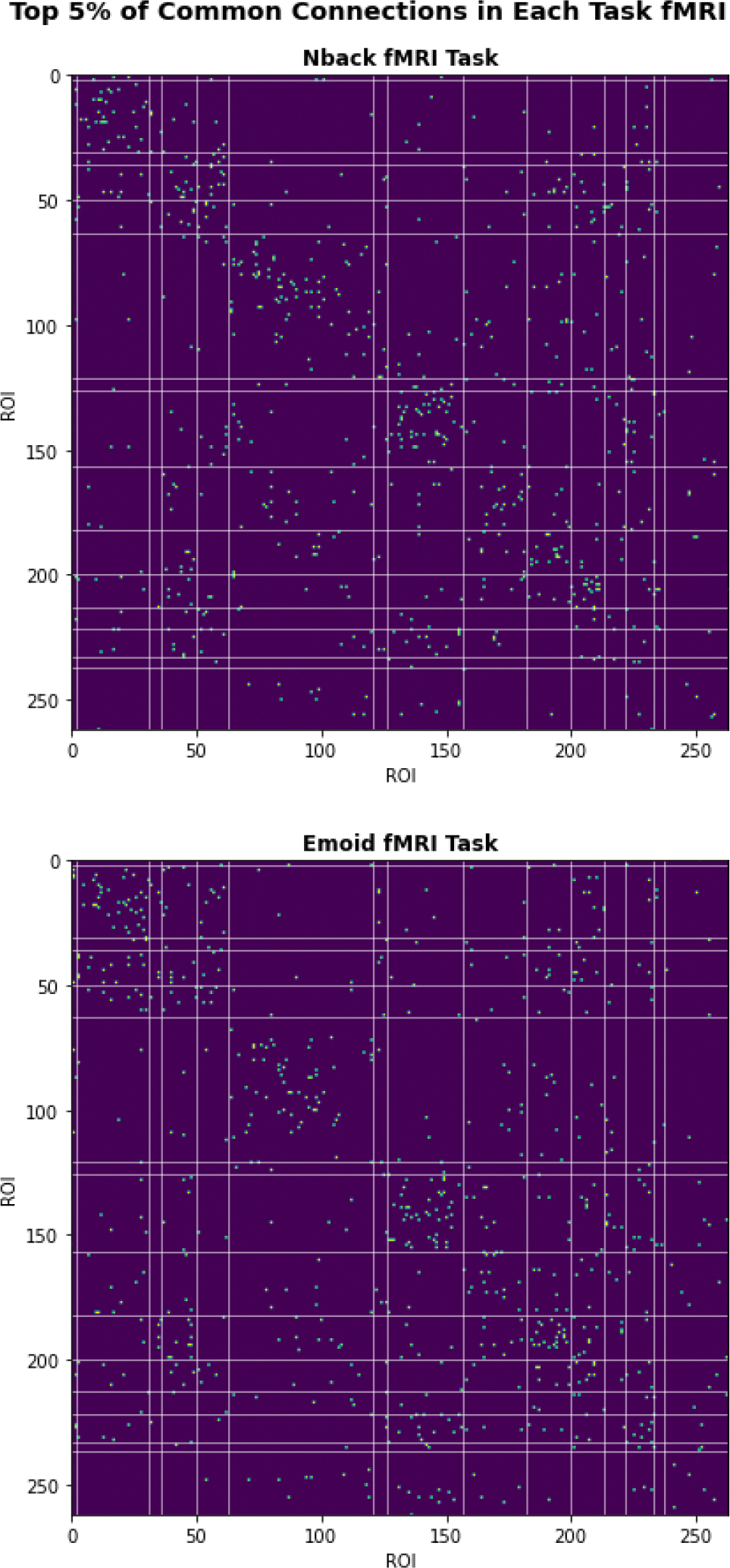
Common connections within each fMRI tasks.

In our study, we introduced an interpretable MJNMF-GAT framework for predicting the age of individuals during different stages of adolescence. We employed both the emoid and nback fMRI tasks to investigate and gain insights into the differences in functional connectivity during different ages during adolescent brain development. To validate our model, we conducted experiments using the PNC dataset and our results indicated that MJNMF-GAT outperformed other baseline models in terms of mean squared error (MSE) and root mean squared error (RMSE). Furthermore, we employed GNNExplainer to interpret our model and identify the significant ROIs and subnetworks responsible for functional connectivity that played a critical role in age prediction. By focusing on the identified important ROIs, we were able to pinpoint the critical subnetworks associated with age prediction. These findings align with previous studies utilizing fMRI, which have also identified age-related differences in functional subnetworks [16], [40], [41]. Fig. 5 depicts the specific number of common connections within each subnetwork during each stage of adolescence.

### A. INTRA-NETWORK CONNECTIONS

By investigating the intra-network connections, we discovered the following differences:

#### 1) DEFAULT MODE NETWORK (DMN)

According to a previous study, the default-mode network (DMN) undergoes alterations with increasing age, specifically exhibiting a consistent decrease in long-range connectivity compared to younger individuals. The primary impact of development on the DMN primarily manifests in the interplay between the medial frontal and posterior midline structures. Furthermore, the functional connectivity between cortical and subcortical nodes within the DMN, such as the hippocampal areas, is also susceptible to age-related alterations, potentially contributing to impaired mnemonic processing. This neurophysiological process associated with development appears to counteract the maturation of functional brain networks, leading to a reduction in short-range connections and the integration of distant regions into cohesive functional networks [42].

#### 2) VISUAL SYSTEM (VIS)

In adolescent neurodevelopment, the visual network becomes more specialized and efficient. Studies have shown increased activation and functional connectivity between visual areas, indicating enhanced integration and coordination within the network. These changes are believed to facilitate improved visual perception, attention, and visual-spatial processing abilities during adolescence. Also, higher-order cognitive control regions, such as the prefrontal cortex, exhibit increased connectivity with visual areas. This enhanced integration between cognitive and visual processing regions supports the development of higher-level visual functions, such as object recognition, attentional control, and decision-making [43].

#### 3) FRONTOPARIETAL TASK CONTROL (FRNT)

Dosenbach et al. investigated the developmental changes in functional connectivity within the FRNT during adolescence and found that the strength of functional connectivity between the prefrontal cortex and parietal regions increased with age, indicating the maturation of network connections within the FRNT. Additionally, the study revealed a strengthening of connectivity between the FRNT and other brain networks involved in sensorimotor processing and cognitive control [44]. Furthermore, Fair et al. examined the developmental changes in functional connectivity within the FRNT and found that during adolescence, there is increased synchronization and integration between the prefrontal cortex and parietal regions, indicating improved coordination and communication within the FRNT [45].

#### 4) SENSORIMOTOR (SM HAND MOUTH)

Uddin et al. investigated the developmental changes in functional connectivity within the sensorimotor network during adolescence, and they found that the strength of connectivity between sensorimotor regions increased with age, indicating the maturation of network connections within the sensorimotor network. The study also revealed a progressive specialization within the network, with increased segregation and distinct functional subnetworks emerging during adolescence [46].

#### 5) MEMORY RETRIEVAL (MEM)

Srokova et al. found that cognitive aging is linked to a diminished capacity for recalling specific details of past experiences. The retrieval-related anterior shift refers to the phenomenon where category-selective cortical activity during retrieval occurs anterior to its peak location during encoding. This shift is more pronounced in older adults compared to younger ones, with evidence showing that a greater anterior shift in the parahippocampal place area correlates with lower memory performance. These findings imply that the anterior shift is sensitive to age-related changes and is influenced by the quality and content of the memory being retrieved [47].

#### 6) SALIENCE (SAL)

Touroutoglou et al. found that connectivity within the ventral salience subsystem not only remained stable but actually intensified with age, while connectivity within the dorsal subsystem diminished over time. Additionally, the age-related enhancement in arousal experience was partially mediated by the increase in ventral salience subsystem connectivity. Additionally, the age-related reduction in executive function was entirely mediated by the decline in connectivity within the dorsal salience subsystem [48].

### B. INTER-NETWORK CONNECTIONS

Additionally, we investigated the inter-network connections, and we found the following differences:

#### 1) MEMORY RETRIEVAL → FRONTOPARIETAL TASK CONTROL

In adolescent neurodevelopment, the memory retrieval fronto-parietal task control network undergoes significant changes during adolescence. Within the memory retrieval network, episodic memory (EM) is believed to play a role in decision-making, particularly by integrating memories into task and goal oriented responses. Therefore, the underdevelopment of EM may contribute to the heightened risk taking behaviors commonly observed during adolescence. Thus, adolescence is a critical period marked by increased susceptibility to the onset of mental health disorders as structural connectivity, functional specialization, and increased integration with cognitive control regions matures [49]. Furthermore, the fronto-parietal task control network also undergoes structural and functional changes, including increased connectivity, enhanced coordination, and improved integration with other brain networks. In adolescence, the FRNT is essential for the development of cognitive control, decision-making, and goal-oriented behavior. This network integrates both internal and external information, enhancing executive functions like working memory and inhibitory control. As FRNT network matures, it plays a key role in modulating impulsive actions and risk taking tendencies [45].

#### 2) FRONTOPARIETAL TASK CONTROL → SALIENCE

During neurodevelopment, functional connectivity between the FRNT and the SAL network enhances the adolescent brain’s ability to detect, interpret, and respond to salient stimuli, thereby facilitating the maturation of higher-order cognitive functions and self-regulation. This interaction is critical for the transition toward more deliberate and controlled behavior characteristic of adulthood [45]. The inter-network connections between the FRNT network and SAL network remain throughout adolescence. Recent research involving the salience network has shown that higher connectivity within SAL is linked to greater sensitivity to both positive and negative peer influences. Adolescents with strong SAL connectivity displayed increased prosocial behavior in response to positive peer norms but were also more likely to engage in risky behaviors when exposed to negative norms. This suggests that the SAL plays a significant role in modulating susceptibility to social influence during adolescence [50].

#### 3) SENSORIMOTOR MOUTH → MEMORY RETRIEVAL

In adolescence, the SM Mouth network undergoes significant changes during adolescence. Sensorimotor systems exhibit increased connectivity between systems and reduced connectivity within systems as individuals age. Previous research suggests the increasing specialization and compartmentalization of sensorimotor networks, which serve to enhance their specific roles in motor control and sensory processing. As development progresses, these systems show a tendency to operate more independently, emphasizing localized processing over integrative interactions with other cognitive networks [51]. Moreover, in adolescence in the MEM network, the maturation of the prefrontal cortex plays a critical role in higher-order cognitive processes and may drive changes in memory retrieval strategies. As adolescents progress toward more efficient and selective cognitive processing, alterations in brain activation patterns during memory retrieval are likely to occur. This shift could support the transition from reliance on concrete, perceptual details to more abstract, conceptual memory retrieval approaches [47].

### C. LIMITATIONS

There are a few limitations in our proposed MJNMF-GAT model for fMRI research. Firstly, in our graph attention network, the features are based on static measures such as the Pearson correlation coefficient, and the edge weights are computed using cosine similarity between functional connectivity features. While these approaches facilitate the learning process, they may not fully capture the dynamic relationships inherent in brain networks. To address this, we plan to explore alternative feature learning techniques and sparsity constraints that could offer a more nuanced representation of edge weights and reduce redundancy in the high-order relationships. Secondly, the current study primarily focuses on identifying significant brain subnetworks and regions of interest related to task-based fMRI data. This approach, while valuable, may overlook the broader context of individual variability in brain function. In future research, we intend to extend our work to analyze differences in brain connectivity across various cognitive tasks and clinical populations. This will help to better understand the generalizability of the model and its applicability to diverse neuroimaging datasets.

## VI. CONCLUSION

In this research endeavor, we proposed an interpretable MJNMF-GAT model for conducting comprehensive analysis of multi-paradigm fMRI data. Our framework demonstrates superior performance in comprehending age differences and discerning significant underlying functional networks as compared to baseline methods. We applied our method to a cohort of brain imaging data to predict individuals by age during the various stages of adolescence. Notably, our model successfully identified crucial age-related functional networks, including DMN, VIS, FRNT, SM Hand Mouth, SAL, MEM, etc. Through a systematic examination of the shared connections among the multi-paradigm data, our study sheds light on significant subnetworks associated with age throughout adolescence. Hence, our proposed method not only contributes to the comprehension of differences in functional connectivity during the various stages of adolescence but also provides valuable insights for early intervention strategies. Importantly, the generic nature of our framework renders it applicable and transferable to the analysis of other imaging data and diverse populations.

## Figures and Tables

**FIGURE 4.**
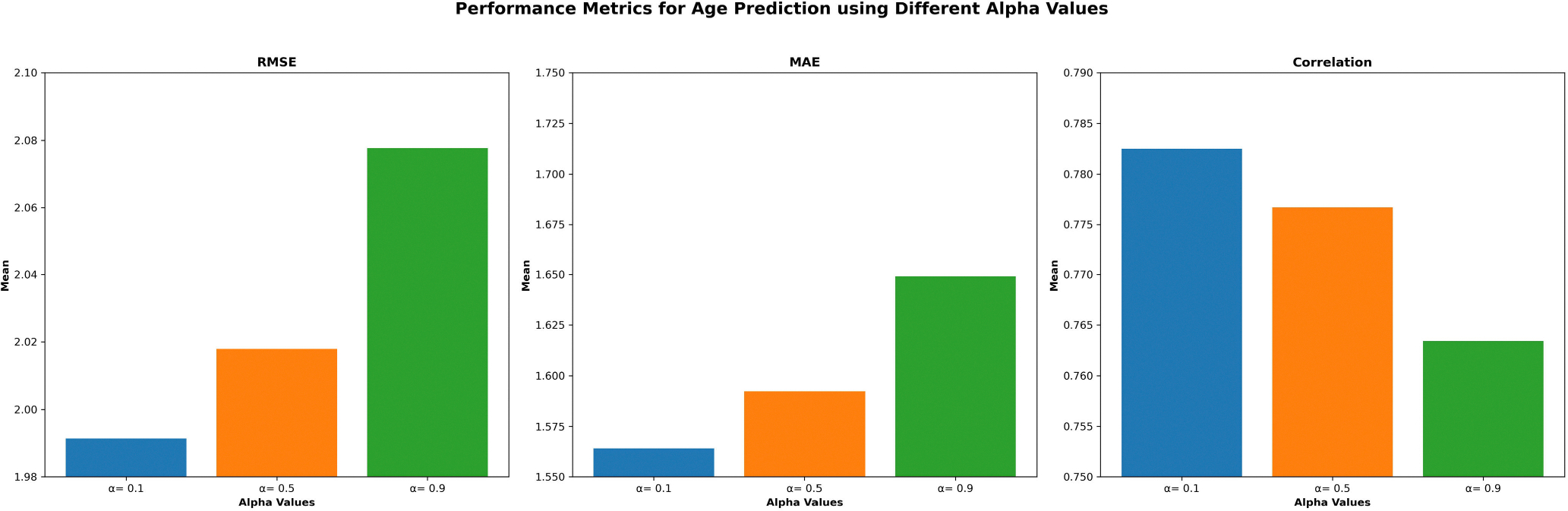
Prediction performance using different alpha values during ablation experiment.

**TABLE 1.** Frequently used notation.

Notation	Description
X	Data matrix representing fMRI features
G=(V,E)	Graph with vertices V and edges E
H	Latent representation matrix obtained from NMF
A	Adjacency matrix of the graph G
F	Feature matrix of graph nodes (brain regions)
W	Weight matrix for graph attention mechanism
Z	Final embedding matrix after graph attention layers
$\alpha_{ij}$	Attention coefficient between nodes i and j
y	Target labels for the fMRI prediction tasks
$\overset{ˆ}{y}$	Predicted labels for each nodes or whole graph
L	Loss function for optimizing JNMF and GAT
λ	Regularization parameter for enforcing sparsity
K	Number of layers in the Graph Attention Network
T	Number of iterations for JNMF factorization

**TABLE 2.** Hyperparameters for GNNExplainer.

Prediction Loss	1
Feature Size Loss	200
Feature Element Loss	20
Population Size Loss	0
Population Element Loss	1000
Weight Decay	0
Training Epochs	150
Learning Rate	5e-1

**TABLE 3.** Hyperparameters for experiments.

Learning rate	1e-5
Optimizer	Adam
Epochs	6000
Layer 1 Size	69,696
Layer 2 Size	100
Weight Decay	0.2
fMRI Paradigms	Emoid, Nback
Predictive Task	Age

**TABLE 4.** Ablation study results: Performance of GAT with and without JNMF.

Model Configuration	RMSE (mean ± std)	MAE (mean ± std)	Corr (mean ± std)
Baseline GAT (No JNMF, Emoid Only)	1.9766 ± 0.1252	1.5536 ± 0.2218	0.7860 ± 0.0494
Baseline GAT (No JNMF, Nback Only)	2.0711 ± 0.4144	1.6543 ± 0.2461	0.7634 ± 0.0545
Baseline GAT (No JNMF, Emoid + Nback)	2.1781 ± 0.2199	1.6688 ± 0.1792	0.7392 ± 0.0592
GAT + JNMF (Rank = 10, *α* = 0.1)	1.9914 ± 0.3261	1.5640 ± 0.2693	0.7825 ± 0.0509
GAT + JNMF (Rank = 10, *α* = 0.5)	2.0179 ± 0.3212	1.5923 ± 0.2791	0.7767 ± 0.0554
GAT + JNMF (Rank = 10, *α* = 0.9)	2.0777 ± 0.5931	1.6491 ± 0.3891	0.7634 ± 0.0543
GAT + JNMF (Rank = 5, *α* = 0.5)	1.9764 ± 0.0761	1.5559 ± 0.2188	0.7862 ± 0.0509
GAT + JNMF (Rank = 20, *α* = 0.5)	1.9820 ± 0.0819	1.5603 ± 0.1224	0.7849 ± 0.0522

* The standard deviation is represented by “std”.

**TABLE 5.** Comparison of regression performance of age prediction by different models.

Model	RMSE (mean ± std)	P-Value	MAE (mean ± std)	P-Value	Corr (mean ± std)	P-Value
LR	2.3432 ± 0.2191	0.0472	1.8414 ± 0.1814	0.0694	0.6843 ± 0.0718	0.0041
MLP	2.0901 ± 0.2009	0.0039	1.6033 ± 0.1734	0.2207	0.7635 ± 0.0559	0.0123
GCN	2.0279 ± 0.1845	0.0122	1.5985 ± 0.1579	0.2362	0.7734 ± 0.0553	0.0081
GAT	2.1781 ± 0.2199	0.0373	1.6688 ± 0.1792	0.1836	0.7392 ± 0.0592	0.0214
MPNN	2.0202 ± 0.1848	0.0378	1.5934 ± 0.1614	0.1496	0.7779 ± 0.0413	0.0526
ONMF	2.0414 ± 0.1714	0.0418	1.6134 ± 0.1621	0.2392	0.7711 ± 0.0273	0.0304
NMF-GCN	2.4282 ± 0.1898	0.0010	1.9349 ± 0.1874	0.0032	0.7004 ± 0.0621	0.0418
PCA-GCN	2.3735 ± 0.1891	0.0021	1.8924 ± 0.1848	0.0053	0.7102 ± 0.0645	0.0351
BrainGNN	2.4231 ± 0.2197	0.0009	1.8934 ± 0.1855	0.0028	0.6540 ± 0.0733	0.0005
BrainNetCNN	2.0123 ± 0.1811	0.0185	1.5783 ± 0.1529	0.1275	0.7824 ± 0.0447	0.0108
MJNMF-GAT*	1.9212 ± 0.1742	-	1.5368 ± 0.1469	-	0.8032 ± 0.0469	-

* The p-values were calculated using a t-test to evaluate the statistical significance of the differences in regression performance between our MJNMF-GAT model and other competing models across repeated experiments.

**TABLE 6.** Top 0.1% of most important connections for age prediction in adolescence.

Modality	ROI	MNI Coordinates (mm)	Anatomical Region	Functional Network (FN)

*Nback-fMRI*	10	10 −46 73	Precuneus R	SM Hand
	17	44 −8 57	Precentral R	SM Hand
	21	−45 −32 47	Postcentral L	SM Hand
	29	38 −17 45	Precentral R	SM Hand
	88	22 39 39	Frontal Sup R	DMN
	101	−20 64 19	Frontal Sup L	DMN
	102	−8 48 23	Frontal Sup Medial L	DMN
	105	−58 −30 −4	Temporal Mid L	DMN
	117	47 −50 29	Angular R	DMN
	126	18 −47 10	Lingual R	VIS
	127	40 −72 14	Occipital Mid R	VIS
	128*	8 −72 11	Calcarine R	VIS
	131	20 −66 2	Lingual R	VIS
	144	42 −66 −8	Temporal Inf R	VIS
	156	37 −81 1	Occipital Mid R	VIS
	168	38 43 15	Frontal Mid R	FRNT
	182	11 −39 50	Cingulum Mid R	SAL

*Emoid-fMRI*	2	−14 −18 40	Undefined	SM Hand
	8	−54 −23 43	Parietal Inf L	SM Hand
	13	29 −39 59	Postcentral R	SM Hand
	35	66 −8 25	Postcentral R	SM Mouth
	90	−10 55 39	Frontal Sup L	DMN
	91	−20 45 39	Frontal Sup L	DMN
	108	13 30 59	Frontal Sup Medial R	DMN
	120	49 35 −12	Frontal Inf Orb R	VIS
	121	−2 −35 31	Cingulum Post L	MEM
	128*	8 −72 11	Calcarine R	VIS
	129	−8 −81 7	Calcarine L	VIS
	132	−24 −91 19	Occipital Mid L	VIS
	139	15 −87 37	Cuneus R	VIS
	146	6 −72 24	Cuneus R	VIS
	167	−42 38 21	Frontal Mid L	FRNT
	173	37 −65 40	Angular R	FRNT
	180	−42 25 30	Frontal Inf Tri L	FRNT
	191	−11 26 25	Undefined	SAL
	192	−1 15 44	Sup Motor Area L	SAL
	193	−28 52 21	Frontal Mid L	SAL
	195	5 23 37	Cingulum Mid R	SAL
	198	26 50 27	Frontal Mid R	SAL
	251	−31 19 −19	Frontal Inf Orb L	UNK
	252	8 −91 −7	Lingual R	UNK
	257	17 −80 −34	Cerebellum Crus 2 R	UNK

MEM: Memory Retrieval, SM: Sensorimotor, DMN: Default Mode Network, VIS: Visual, FRNT: Fronto-parietal Task Control, SAL: Salience, UNK: Unknown
